# Imaging Patterns of Temporal Bone Fracture among Patients with Head Injury at Tikur Anbessa Specialized Hospital, Ethiopia

**DOI:** 10.4314/ejhs.v33i6.8

**Published:** 2023-11

**Authors:** Elsabeth Wondwossen Yimam, Amal Saleh Nour, Tequam Debebe, Tewodros Endale

**Affiliations:** 1 Jimma University, College of Public Health and Medical Sciences, Department of radiology, Jimma, Ethiopia; 2 Addis Ababa University, College of Health Sciences, Department of Radiology, Addis Ababa, Ethiopia

**Keywords:** Fracture, Head injury, Imaging patterns, Temporal bone, Trauma

## Abstract

**Background:**

Temporal bone fracture is usually a sequel of significant blunt head injury. Fracture of the temporal bone is mainly classified according to the orientation of the fracture plane and whether there is involvement of the otic capsule. Despite its frequent occurrence, there is limited research on the frequency and pattern of temporal bone fractures in our setup.

**Methods:**

Retrospective cross-sectional hospital - based study of 60 patients who underwent computed tomography of the head for head trauma at Tikur Anbessa Specialized Hospital during the study period from October 2020 – October 2022.

**Results:**

Among the 60 patients enrolled in the study, the mean age of presentation was 31.1 years with a male-to-female ratio of 4:1. There were 69 temporal bone fractures, 9(15%) were bilateral and 51(85%) unilateral The longitudinal fracture pattern was the most common fracture pattern, occurring in 40(78.4%) of unilateral cases, 15(83.3%) of bilateral cases. Otic capsule sparing fractures accounted for 49(96.07%) of unilateral fracture cases, and all patients with bilateral involvement had an otic capsule sparing fracture. Among the 42 patients for whom data regarding post-traumatic hearing outcome was available, 4 patients had post-traumatic hearing impairment. Anatomically, the squamous portion of the temporal bone was involved in 30(43.5%) of cases.

**Conclusions:**

Fractures affecting the squamous portion of the temporal bone, longitudinal fracture patterns, and otic capsule sparing were the most frequent forms. The majority of temporal bone fractures were associated with other bone fractures and intracranial injuries.

## Introduction

Trauma, sometimes called the silent epidemics, is the most common worldwide cause of death in children and young adults. Neuro trauma is responsible for the vast majority of these cases. The temporal bones are located on the lateral aspects of the skull, beneath the parietal bones, behind the sphenoid bone, and in front of the occipital bone. In addition to their role in the formation of the neurocranium, the temporal bones also contribute to the formation of the middle and posterior cranial fossae. The temporal bone has five parts. In addition to its main function of shielding the brain, each temporal bone has several critical structures including the apparatus of the middle and internal ears. Temporal bone fractures and their complications may be easily identified and characterized on cervical, maxillofacial and head multidetector CT performed in patients with polytrauma, without the need for dedicated temporal bone CT (1-4).

A fracture of the temporal bone usually results from a significant blow to the head, and a penetrating injury to the temporal bone is uncommon. Associated intracranial injuries, such as extra-axial hemorrhage, diffuse axonal injury and cerebral contusions are common. Furthermore, potential hearing impairment and facial nerve damage may occur (5). The complications of temporal bone fracture include facial nerve involvement (facial nerve palsy), disruption of the ossicular chain, involvement of the otic capsule (vertigo and sensorineural hearing loss), disruption of cerebrospinal fluid (CSF) (CSF otorrhea or CSF rhinorrhea and meningitis), post-traumatic cholesteatoma, and perilymphatic fistula (5).

In the majority of occurrences, fractures involving the petrous temporal bone is classified according to the primary orientation of the fracture plane and whether or not the otic capsule is involved. Fractures of the temporal bone are described in relation to the long axis of the petrous temporal bone, which runs obliquely from the petrous apex posterolaterally through the mastoid air cells. This axis is determined by the orientation of the fracture. When viewed from this perspective, fractures can be categorized as longitudinal, transverse, or mixed fractures (5-7).

Although a fracture of the temporal bone is one of the most common causes of morbidity, to the best of our knowledge, no research has been conducted to determine how often temporal bone fractures occur or what patterns they take in our setup. Therefore, the goal of this study was to determine the pattern of temporal bone fractures among patients who had CT scans of the head performed at TASH in Ethiopia during the years 2020–2022.

## Material and Methods

**Study area and period**: The study was conducted at Tikur Anbessa Specialized Hospital (TASH) during the study period of October 2020-October 2022GC. TASH is the largest tertiary referral teaching university Hospital. TASH is one of the largest hospitals found in the nation's capital city, Addis Ababa. It is one of the largest referral centers in the country and the main teaching hospital. The hospital provides a tertiary-level referral treatment with over 900 beds and is open 24 hours a day for emergency services.

**Study design**: An institution-based retrospective cross-sectional study design was employed on all patients who underwent CT scan of the head for head trauma at TASH from October 2020-October 2022GC.

**Study population**: All patients who underwent CT scan of the head for head trauma at TASH and had temporal bone fracture during the study period, October 2020-October 2022GC.

**Sampling technique and sample size**: Due to the lack of research on this topic in our country, the sample size was calculated using a single population proportion calculation with a p-value of 50% and a 95% confidence interval. The final sample size became 138 after a small population correction and an assumption of 15% chart loss. However, because the number of patients with temporal bone fracture was small, all charts were reviewed.

**Data collection and analysis**: Important patient data including the demographic data, and the clinical conditions of the patient including the patient presentation were collected from patients' chart using a structured questionnaire and exported to statistical package for social sciences (SPSS) version 26. Statistical analysis was performed. Analysis using mean, summary tables, chi square test, and P-values less than 0.05 were considered statistically significant. Head CTs of patients who fulfilled the inclusion criteria were reviewed using the open-source DICOM viewer. The following operational definitions were used:

**Traditional classification**: classification system that classifies temporal bone fractures into longitudinal, transverse and oblique fracture patterns

**Longitudinal fractures**: petrous temporal bone fractures that occur parallel to the long axis of the petrous temporal bone

**Transverse fractures**: temporal bone fractures that run perpendicular to the petrous bone's long axis and extend toward the jugular foramen and temporal fossa

**Mixed or Oblique Fractures**: complex fractures that are difficult to classify as longitudinal or transverse because they have both longitudinal and transverse elements

**New classification (otic capsule sparing or otic capsule involving)**: classification schemes which classify temporal bone fractures in to otic capsule sparing or otic capsule involving

**Otic Capsule Involving fracture**: the fracture line passes through the Otic capsule

**Otic capsule sparing fractures**: the fracture line runs anterolateral to the otic capsule, involving the squamous portion of the temporal bone, the external auditory canal, the tympanic membrane, which is frequently involved, as well as the mastoid air cells and middle ear

**Ethical consideration**: Ethical approval was obtained from the Ethical Review Committee of the Department of Radiology of the College of Health Sciences of Addis Ababa University. By avoiding using patient identifiers in the database, patient confidentiality was maintained. The patient records were accessed with official consent.

## Results

**Figure 1:**
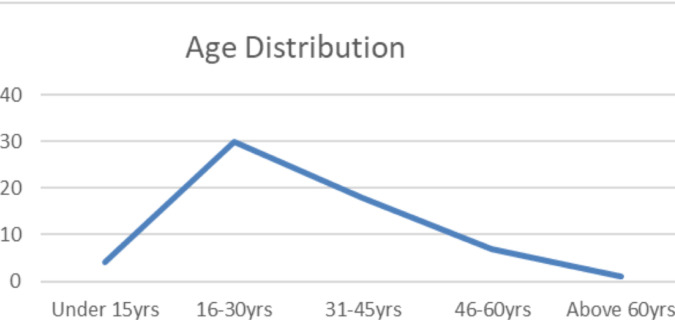
Age distribution of 60 patients with temporal bone fracture at TASH in the 2020-2022 G.C., Addis Ababa, Ethiopia

Of the 1395 head injury patients who had a CT scan of the head, 60 patients (4.3%) who had temporal bone fracture were enrolled in the study group. Forty-eight (80%) were male and 12 (20%) were female, with a male-to-female ratio of 4:1. The mean age of presentation was 31.1 ± 13.87 years (Fig. 1).

There was a record of their mechanism of injury in 58(96.6%) of the patients, which were found to be road traffic accident (RTA) and falling accident, representing 38(63.3%) and 11(18.3%) of the study participants, respectively. In terms of clinical presentation, the patients had a history of loss of consciousness, otorrhea, and rhinorrhea, accounting for 27(45%), 17(28.3%), and 9(15%) of the total. At the time of presentation, 3(5%) of the patients had facial nerve palsy (Table 1).

**Table 1:** Sociodemographic profile and clinical presentation data of 60 patients with temporal bone fracture at TASH in 2020-2022 G.C., Addis Ababa, Ethiopia (n=60)

Variables	Frequency (%)
**Sex**	
Male	48 (80%)
Female	12 (20%)
**Age**	
Under 15yrs	4 (6.7%)
16-30yrs	30 (50%)
31-45yrs	18 (30%)
46-60yrs	7 (11.7%)
Above 60yrs	1 (1.7%)
Range	2-62yrs
Mean	31.1yrs
**Mechanism of injury**	
Road traffic accident	38 (63.3%)
Falling down accident	11 (18.3%)
Stick injury	4 (6.7%)
Stone injury	2 (3.4%)
Blast injury	1 (1.7%)
Bullet injury	1 (1.7%)
Domestic violence	1 (1.7%)
Unspecified	2 (3.4%)
**Clinical presentation**	
Loss of consciousness	27 (45%)
Otorrhea	17 (28.3%)
Rhinorrhea	9(15%)
Facial nerve palsy	3 (5%)
Hearing impairment	2 (3.3%)

**Figure 2:**
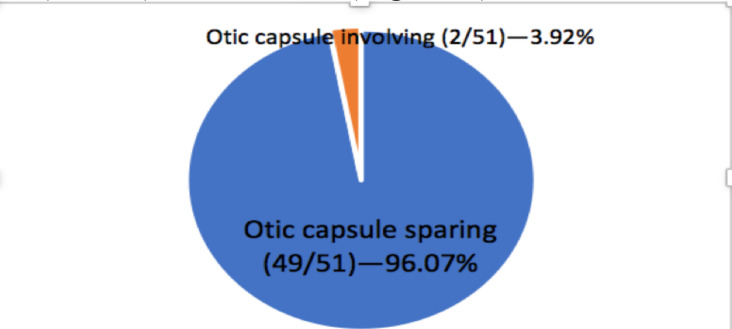
Types of temporal bone fracture based on the new classification system of patients with temporal bone fracture at TASH in 2020-2022, Addis Ababa, Ethiopia

Among the 60 patients, there were 69 temporal bone fractures with 9(15%) being bilateral and 51(85%) unilateral. The most common morphologic fracture pattern was the longitudinal fracture pattern in 55(79.7%) of the patients. In addition, a significant majority of the fractures were otic capsule-sparing, which represented 67(97.1%) of the cases (Figure 2).

**Figure 3:**
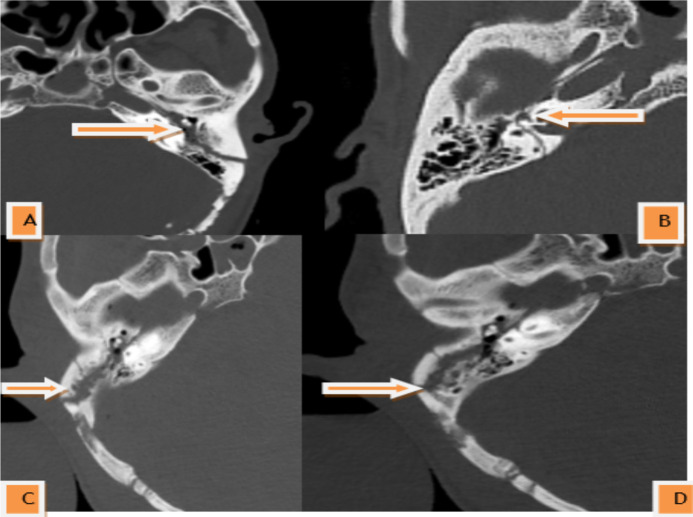
Axial non-contrast bone window CT scan of the head of three different trauma patients showing types of temporal bone fracture based on traditional classification system. Longitudinal fracture(A) transverse fracture (B) and mixed fracture C & D

**Figure 4:**
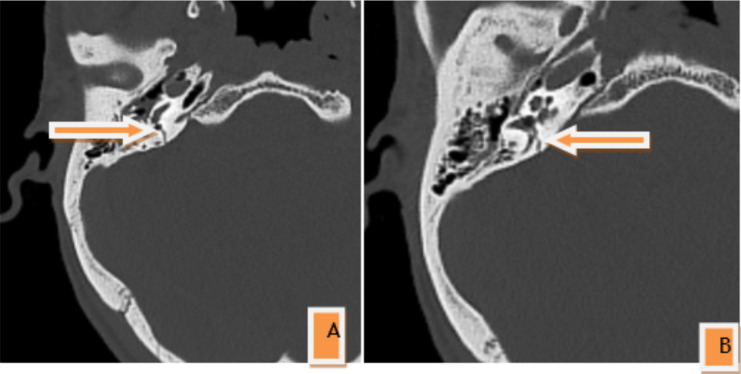
Axial non contrast bone window CT scan of a 35 years old male patient showing Right petrous temporal bone, otic capsule violating transverse fracture with involvement of the right ICA(A) and jugular fossa walls (B)

If the bilateral cases are taken into consideration separately, the longitudinal fracture pattern was the most common fracture pattern, occurring in 40(78.4%) of the unilateral cases, 15(83.3%) of the bilateral cases. Otic capsule sparing accounted for 49(96.07%) of the unilateral fracture cases, and all patients with bilateral involvement had an otic capsule sparing fracture (Figure 3, Figure 4, Table 2).

**Table 2:** Imaging classification patterns of 60 patients with temporal bone fracture using Head CT done at TASH in the 2020-2022, Addis Ababa, Ethiopia

Variables		Frequency (%), n=60	
**Laterality**			
	Left only	24 (40%)	
	Right only	27 (45%)	
	Both	9 (15%)	
**Traditional classification system**		Left side (n=24)	Right side (n=27)
**Unilateral**	Longitudinal (40/51)—78.4%	18	22
	Transverse (8/51)—15.6%	5	3
	Mixed/Oblique (3/51)—5.8%	1	2
**Bilateral**	Longitudinal (15/18) —83.3%	7	8
	Transverse (2/18) —11.1%	1	1
	Mixed/Oblique (1/18) —5.5%	1	0
**Anatomic classification system**			
	Involving the Squamous Part (30/69)—43.5%	14	16
	Involving the Mastoid part (part (10/69)—14.5%	4	6
	Involving the Petrous part (6/69)—8.7%	2	4
	Involving the Tympanic part (3/69)—4.4%	1	2
	Mixed Type (20/69)—28.9%	12	8

Anatomically, the squamous portion of the temporal bone was involved in 30(43.5%) of cases followed by mixed type in which different combinations of the temporal bone parts were involved in 20(28.9%) cases. The third most commonly involved among the isolated parts of the temporal bone was the mastoid part in 10 (14.5%) of the cases (Table 2).

**Figure 5:**
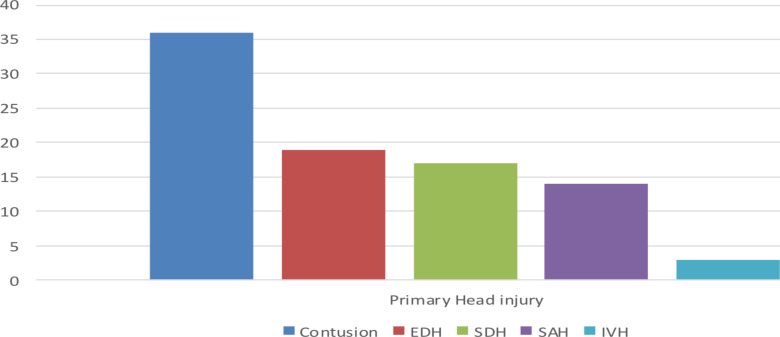
Concomitant primary head injury in patients with temporal bone fracture with head CT done at TASH in 2020-2022, Addis Ababa, Ethiopia

With regards to other associated injuries, there were other bone fractures in 56(93.3%) cases and intracranial injuries in 48(80%) of the patients. The skull vault bone fractures accounted for 47(83.9%) of the cases, followed by contusions, which involved 19(39.5%) of the cases (Figure 5).

Among the 60 patients in our study, 42 had a record of the presence or absence of post-traumatic hearing impairment in their charts, of which 4(9.5%) had post-traumatic hearing impairment. Three of them had conductive hearing loss, and one patient had a mixed type of hearing loss (Table 3). Among the 4 patients who had post-traumatic hearing impairment 3(75%) had otic capsule sparing fracture, and 1(25%) had otic capsule violating fracture.

**Table 3:** Post-traumatic hearing impairment in 60 patients with temporal bone fracture with head CT doneat TASH in the years 2020-2022 G.C., Addis Ababa, Ethiopia

Post Traumatic hearing impairment	N=42
Yes	4 (9.5%)
No	38 (90.4%)
Non-Specified	18 (30%)
**Type of Hearing Loss**	**N=4**
CHL	3 (75%)
Mixed	1 (25%)
SNHL	0

Among the 3 patients who had post-traumatic CHL, 2 had longitudinal fracture while 1 had transverse fracture, and all of the fracture components involved the mastoid part of the temporal bone. The patient with the transverse fracture also had incudo-malleolar joint disruption, and one patient had an incus fracture.

## Discussion

In our study, out of 1395 who had CT, 60(4.3%) of the head injury patients had temporal bone fracture. This is in line with a study by Z Amin and colleagues at the Department of Otorhinolaryngology-Head and Neck Surgery, which involved 1309 patients who had suffered head injuries and found that 61 of them had temporal bone fractures (4.7%) (8). However, research by Dahiya R. and colleagues at the University of Massachusetts Medical School, which involved 2,977 head trauma patients reported that the incidence of temporal bone fracture was 90 (3%), which is slightly lower than our findings. This can be attributed to the higher prevalence of road traffic accidents in our country, TASH being one of the biggest referral hospitals in Addis Ababa, and the small sample size of our study (9).

Our study showed that the most commonly affected patients with temporal bone fracture with regard to sex and age are males in the age ranges of 16-45yrs. The average age was 31.1 years which is consistent with most studies (10-12).

The most common clinical presentations of the patients were loss of consciousness, CSF leak, and facial nerve palsy which is consistent with a study done in Ohio at the University of Cincinnati, whose study showed that the most common clinical presentations of the patients were CSF leak and facial nerve palsy (13). Most of the studies showed that the majority of temporal bone fractures were caused by RTA. This is comparable with our study, which showed that the prevalence of head injuries resulting from RTA was 38 (63.3%), which emphasized the ongoing issue of the high incidence of RTA in our nation (14).

Anatomically, the most commonly fractured portion of the temporal bone was the squamous part; a study done in Ohio also showed that non-petrous temporal bone fractures are much more common than petrous TBFs (9). The majority of temporal bone fractures in our study were unilateral, with isolated left and right side fractures of 40% and 45%, respectively. Only 15% of patients had bilateral fracture, which is consistent with most studies (14).

Most studies showed that the longitudinal pattern of fracture is the most common fracture pattern (6-8,10). Our study also showed that the longitudinal fracture is the most common temporal bone fracture pattern, representing 78.4% of the unilateral fractures. The majority of temporal bone fractures, however, are mixed fractures rather than pure longitudinal or transverse fractures according to a study done at the University of Texas Health Science Center in Houston. Most of the fractures had both longitudinal and transverse components and are therefore classified as mixed fractures rather than as either one of the two types of fracture exclusively (11).

With regards to the new classification system, we found that otic capsule sparing fractures accounted for the vast majority of the fracture patterns representing 96.07% of the fractures. In other studies, otic capsule-sparing fractures were also more common than otic capsule violating fractures, even though the figures were lower in the range of 80–85% (7,8,10,15,16).

Forty-eight (80%) of the patients had an associated primary type head injury, of which contusion was the most common followed by an epidural hematoma. This can be attributed to the tremendous amount of force required to fracture the temporal bone, the complex anatomy, and many critical structures in close proximity. In a prospective study by Padmakumar V that included 2748 polytrauma cases and looked at the relationship between temporal bone involvement in those patients and the effect of early diagnosis on hearing loss, 90 people (or 3.2% of the total) had temporal bone fractures, and 60 patients (66.7%) had intracranial injuries associated with temporal bone fractures. This is consistent with our findings (14).

Our study showed that, of those with recorded data for hearing impairment, post-traumatic hearing impairment occurred in 4(9.5%) patients. However, a study done on the current perspective on temporal bone trauma in San Francisco, California, involving 34 patients with 44 fractures, showed that 15 (44.1%) of the patients had hearing impairment (7).

Another study was done in India on 278 polytrauma patients, and the result showed that 90(3.2%) of the patients had temporal bone fracture, of which 56(62.2%) had hearing impairment (14). This can be attributed to the fact that there was no documentation of hearing outcome recorded on patient charts for 18(30%) patients which could in turn affect the true findings of patients in our study.

In conclusion, the most common morphologic pattern is the longitudinal fracture pattern with a significant majority of fractures being otic capsule sparing. RTA was the most frequent injury mechanism with loss of consciousness and otorrhea being the commonest clinical presentation as well as associated intracranial injuries. The majority of the patients had no mention of hearing outcome in their charts. We recommend awareness creation on the importance of documenting and assessment of hearing impairment in patients with temporal bone fractures is vital as hearing loss is a cause of significant morbidity.

## References

[1] Osborn AG, Hedlund GL, Salzman KL (2018). Osborn's brain: imaging, pathology, and anatomy.

[2] Zayas JO, Feliciano YZ, Hadley CR, Gomez AA, Vidal JA (2011). Temporal bone trauma and the role of multidetector CT in the emergency department. Radiographics.

[3] Hoeffner EG, Mukherji Suresh Kumar, Gandhi D (2011). Temporal Bone Imaging.

[4] Butler P, Mitchell A, Healy JC (2012). Applied Radiological Anatomy.

[5] Som PM, Hugh D (2011). Curtin MD Head and neck imaging 1.

[6] Schubiger O, Valavanis A, Stuckmann G, Antonucci F (1986). Temporal bone fractures and their complications. Examination with high resolution CT. Neuroradiology.

[7] Dreizin D, Sakai O, Champ K, Gandhi D, Aarabi B, Nam AJ (2021). CT of Skull Base Fractures: Classification Systems, Complications, and Management. RadioGraphics.

[8] Amin Z, Sayuti R, Kahairi A, Islah W, Ahmad R (2008). Head injury with temporal bone fracture: one year review of case incidence, causes, clinical features and outcome. Med J Malaysia.

[9] Dahiya R, Keller JD, Litofsky NS, Bankey PE, Bonassar LJ, Megerian CA (1999). Temporal bone fractures: otic capsule sparing versus otic capsule violating clinical and radiographic considerations. J Trauma.

[10] Johnson F, Semaan MT, Megerian CA (2008). Temporal bone fracture: evaluation and management in the modern era. Otolaryngol Clin North Am.

[11] Ghorayeb BY, Yeakley JW (1992). Temporal bone fractures: longitudinal or oblique? The case for oblique temporal bone fractures. Laryngoscope.

[12] Ricciardiello F, Mazzone S, Longo G, Russo G, Piccirillo E, Sequino G (2021). Our Experience on Temporal Bone Fractures: Retrospective Analysis of 141 Cases. J Clin Med.

[13] Ishman SL, Friedland DR (2004). Temporal bone fractures: traditional classification and clinical relevance. Laryngoscope.

[14] Padmakumar V, Ramesh Kumar E, Ramakrishnan VR (2020). A Prospective Study on Temporal Bone Involvement in Polytrauma Patients and the Effect of Early Diagnosis on Hearing Loss. Indian J Otolaryngol Head Neck Surg.

[15] Little SC, Kesser BW (2006). Radiographic classification of temporal bone fractures: clinical predictability using a new system. Arch Otolaryngol Head Neck Surg.

[16] Nosan DK, Benecke JE, Murr AH (1997). Current perspective on temporal bone trauma. Otolaryngol Head Neck Surg.

